# The Association of Deployment-related Probable Traumatic Brain Injury with Subsequent Medical Readiness Status

**Published:** 2025-01-20

**Authors:** Andrew J. MacGregor, Amber L. Dougherty, James M. Zouris, Sarah M. Jurick

**Affiliations:** 1 Naval Health Research Center, San Diego; 2 Leidos, Inc., San Diego

## Abstract

Traumatic brain injury (TBI) has been a major source of morbidity within military forces during the last 2 decades, but research on the relationship between TBI and medical readiness is limited. This study population included 41,442 service members from the U.S. Navy and Marine Corps who completed a Post-Deployment Health Assessment (PDHA) and a Periodic Health Assessment (PHA). Presence of TBI was ascertained from a screening instrument on the PDHA, and provider determination of medical readiness was abstracted from the PHA. Multivariable logistic regression assessed the association between probable TBI and ‘not medically ready’ (NMR) service member disposition while adjusting for covariates. Overall, 1.8% of the study population screened positive for TBI, and individuals with TBI had a significantly higher prevalence of NMR disposition (7.8%) than those without (3.7%). After adjusting for all covariates, TBI was associated with higher odds of post-deployment NMR disposition (odds ratio 1.5; 95% confidence interval, 1.2-2.0). Deployment-related TBI is associated with medical readiness. Future studies are needed to elucidate the TBI sequelae that may lead to NMR disposition as well as the impact of repeated TBIs.

**What are the new findings?:**

This study identified 54% increased odds of ‘not medically ready’ disposition for military personnel with probable traumatic brain injury (TBI) following deployment, after adjusting for post-traumatic stress disorder and other covariates.

**What is the impact on readiness and force health protection?:**

This analysis measures the association of TBI with medical readiness, which could inform future TBI screening, referral, and patient management protocols. Awareness of this association is particularly important in times of high operational tempo, during which maintaining force readiness through multiple deployment cycles is imperative.

## 1 BACKGROUND

Traumatic brain injury (TBI) is a prevalent condition in the U.S. military. It is estimated that as many as 1 in 5 service members experienced possible TBI during combat deployment in Iraq and Afghanistan.^1^ This estimate was primarily attributed to the widespread use of blast weaponry by the enemy, which caused more than 70% of combat casualties.^2^ New research suggests that the risk of TBI among military personnel may be even higher in conflicts with “near peer” adversaries and more advanced weapons.^3^

Besides combat operations, TBI can also occur in military personnel as a result of training, accidents, sports activities, and occupational exposure (e.g., low-level blasts).^4,5,6,7^ The TBI Center of Excellence reported 485,553 TBI incidents between 2000 and the second quarter of 2023.^8^ TBI is an important medical condition for military leadership to consider during times of war and peace.

Sequelae of TBI include neurological, physical, sensory, and psychological complaints that can often co-occur, sometimes leading to multimorbidity.^9,10^ This includes a strong association with post-traumatic stress disorder (PTSD), an anxiety disorder resulting from exposure to a traumatic event, which can complicate TBI treatment.^11^ TBI among military personnel may have an extended symptom course compared to the anticipated recovery trajectory,^12^ even showing a deleterious effect on quality of life several years after the injury.^13^

Health care costs associated with TBI are also significant. A recent study estimated that the median cost of combat-related TBI during the first year after injury was $129,655 for moderate-to-severe TBI and $74,810 for mild TBI,^14^ although this did not account for the financial impact of TBI on readiness (i.e., ability of military personnel to deploy in support of operations). With an all-volunteer force and recent lags in recruiting,^15^ maintaining medical readiness among current military service members is essential.

There is a gap in the literature on the association between TBI and medical readiness, and quantifying this issue is essential for advising medical planners, clinicians, and policy-makers. The objective of this study was to examine the association between deployment-related TBI and post-injury not medically ready (NMR) disposition. Two standardized health questionnaires, the Post-Deployment Health Assessment (PDHA) and the Periodic Health Assessment (PHA), were used to identify TBI and NMR disposition, respectively.

## 2 METHODS

Data were abstracted from the PDHA and PHA for service members in the Navy and Marine Corps. The PDHA is a screening questionnaire given to personnel at the conclusion of deployment, with questions on a variety of deployment-related health issues including TBI and PTSD.^16^ The PHA is an annual health questionnaire for all service members that features a provider determination of medical readiness status.^17^

For personnel with at least 1 completed PDHA (DD FORM 2796, Oct. 2015) between January 1, 2016 and December 31, 2019, the most recent PDHA was selected. These data were linked to the first PHA (DD FORM 3024, Apr. 2016) that was completed and certified by a health care provider within 18 months after the completion of the selected PDHA. This methodology resulted in an initial study population of 42,914. A total of 1,472 personnel were excluded from analysis due to missing data for independent or dependent variables, leaving a final study population of 41,442.

Presence of TBI was measured using a PDHA screening instrument that is based on the Brief Traumatic Brain Injury Screen.^18^ This screening instrument asks respondents if they experienced an injury event (e.g., blast or explosion, vehicular accident or crash, fragment or bullet wound, or other injury) and whether that injury event resulted in a loss or alteration of consciousness. Those endorsing both an injury event and a loss or alteration of consciousness were classified as ‘TBI screen positive’, i.e., probable TBI. PTSD was also measured on the PDHA using the Primary Care PTSD Screen, on which endorsement of 2 of the 4 PTSD questions indicated a PTSD screen positive.^19^

NMR disposition and all other covariates were abstracted from the PHA. Presence of NMR disposition was obtained from the Individual Medical Readiness Disposition Determination section on the PHA completed by a health care provider. NMR is defined on the PHA as “Service members with a chronic or prolonged deployment-limiting medical or mental condition as described in DoDI 6490.07.”

Time between each PDHA and PHA was calculated in days and entered as a continuous variable for analysis. Age was categorized in years, as 18-24, 25-29, 30-34, 35-39, and 40 and older, sex was categorized as male or female, service as Navy or Marine Corps, military rank as enlisted or officer, and service component as active or reserve/national guard.

Univariate analysis examined NMR disposition and all covariates for those with and without probable TBI, using Chi-square and t-tests for categorical and continuous variables, respectively. Multivariable logistic regression assessed the relationship between probable TBI and NMR disposition while adjusting for all covariates. Odds ratios (ORs) and 95% confidence intervals (CIs) were reported. The Hosmer-Lemeshow test was used to evaluate model fit with an alpha level of 0.1. All analyses were conducted in SAS version 9.4 (Cary, NC).

## 3 RESULTS

Descriptive statistics are presented in **Table 1**. The study population was primarily younger than 30 years old (61.4%), male (90.2%), in the Marine Corps (58.8%), enlisted (83.2%), and active duty (79.7%) at time of PHA. The prevalence of PTSD positive screenings and NMR disposition was 4.6% and 3.7%, respectively; on average, the PHA was completed 239 days (SD 150) after the PDHA.

Overall, the prevalence of screening positive for TBI was 1.8% (760/41,442). All variables, except rank and time between PDHA and PHA, differed significantly by probable TBI. Individuals with probable TBI were more likely than those without to be older, female, in the Navy, and reserve or national guard. Most notably, those with probable TBI (27.6%) had a significantly higher prevalence of probable PTSD than those without TBI (4.1%). NMR disposition was significantly higher (7.8%) in military personnel with probable TBI compared with those without TBI (3.7%; p<0.001).

Results from the multivariable logistic regression are shown in **Table 2**. Service members who screened positive for TBI on the PDHA, compared with those who did not, were significantly more likely to have a post-injury NMR disposition on the PHA (OR 1.5; 95% CI, 1.2-2.0). The strongest associations with NMR disposition were a positive screening for PTSD (OR 2.5; 95% CI, 2.1-2.9), female sex (OR 1.9; 95% CI, 1.6-2.2), and reserve or national guard status (OR 2.0; 95% CI, 1.8-2.2). The Hosmer-Lemeshow test indicated the model as a good fit (p>0.10).

## 4 DISCUSSION

In this analysis, probable deployment-related TBI was associated with 54% increased odds of post-injury NMR disposition among Navy and Marine Corps personnel. This finding has implications for military medical and operational planning, and future research is needed to determine the effects of specific TBI sequelae on this relationship. We identified associations between a positive screen for PTSD and both probable TBI and NMR disposition, which emphasizes the need to account for PTSD when studying TBI and related outcomes among military personnel.

The prevalence of TBI in the present study was only 1.8%, which is lower than in earlier studies from the conflicts in Iraq and Afghanistan in which TBI screen positive rates ranged from 15% to 23%.^20,21,22^ This difference may be attributable to operational tempo during the study period, whereby the present analysis excluded earlier years of the post-9/11 overseas contingency operations that were characterized by high operational tempo and injury rates.^23,24^ All deployments were also included in this study, rather than only those to a combat zone, as in the previous research. Altogether, this suggests the effect of probable deployment-related TBI on medical readiness may be greater at times of high operational tempo, when there may be an increased risk of injury, which could be exacerbated by protracted operations in which military personnel are expected to deploy multiple times to a combat zone. Epstein et al. (2023)^3^ posited that TBI may occur at a greater incidence in future conflicts against ‘near peer’ adversaries, which makes the study of TBI and medical readiness even more critical.

It is important to determine the specific symptoms of TBI that may drive the relationship between deployment-related TBI and post-deployment NMR disposition, as this information could be used to refine screening and referral protocols, as well as patient management guidelines, to maximize medical readiness. Previous research identified associations between specific TBI symptoms and health-related outcomes. For example, post-TBI dizziness and memory problems were associated with declines in self-rated health.^25^ Further research is needed to examine the independent effect of deployment-related TBI symptoms on readiness. The 2008 form of the PDHA included questions on the TBI screening instrument that queried service members on current symptoms related to a TBI, but this section was removed on the 2012 form revision and has not been included in subsequent revisions. Nevertheless, the PDHA contains a generalized checklist of symptoms that could be used for this analysis, and data reduction techniques such as latent class or cluster analysis could be employed to identify symptom profiles following deployment-related TBI associated with decreased readiness.^10^

PTSD was the strongest predictor of NMR status in this study. Not surprisingly, PTSD was also associated with TBI, which is consistent with previous literature.^11^ It is important to note that many TBI symptoms overlap with PTSD,^21^ and prior research has shown that, after accounting for PTSD, the relationship between TBI and several self-reported TBI symptoms is attenuated. For example, in one study of military personnel, Hoge et al. (2008)^20^ found that only headache was independently associated with TBI after adjusting for PTSD, and other studies have yielded similar findings.^21,25^ As such, it is important to account for PTSD and potential symptom overlap with TBI while examining the short- and long-term impacts of TBI in military populations. Furthermore, clinicians should consider PTSD when treating military patients with TBI and incorporate multidisciplinary care if necessary to ensure a whole health treatment approach.

The primary strength of this study was the ability to link PDHA with PHA data, which allowed for a direct assessment of medical readiness in a post-deployment population. Using all available data for the Navy and Marine Corps resulted in a robust sample size and ability to detect statistically significant associations when examining NMR disposition, a relatively infrequent event. There were also several limitations. The study population was restricted to only those who deployed and completed both a PDHA and PHA, which may affect the generalizability of the results, as non-deployed service members were not included. These results may also not be generalizable to conflicts during times of high operational tempo. In addition, compliance with the questionnaires can vary. Furthermore, both TBI and PTSD were assessed with a screening instrument, which is not the ‘gold standard’ approach and cannot parse TBI severity (e.g., mild versus moderate).

The medical readiness disposition determination section on the PHA does not collect information on specific conditions leading to an NMR designation, so it was impossible to determine whether NMR disposition was assigned because of deployment-related TBI. Finally, this study did not assess the effects of multiple deployments, repeated TBI events, or pre- and post-deployment medical conditions, and additional research is needed to account for these factors.

Medical readiness is an important, ongoing issue for the military, and this study indicates that deployment-related TBI is associated with adverse readiness outcomes. Future studies should focus on the relationship between specific TBI sequelae and medical readiness while accounting for co-occurring conditions, such as PTSD, with similar symptomologies. As TBI continues to burden the U.S. military, further research is needed to improve the identification and management of these injuries to ensure individual and force readiness.

## Figures and Tables

**Table 1:** Characteristics of the Study Population by Traumatic Brain Injury Screening Status

Characteristic	Total		TBI Positive Screen		TBI Negative Screen		*P*-value
	(n=41,442)		(n=760)		(n=40,682)		*P*-value
	No.	%	No.	%	No.	%	
Age, y							<0.001
18-24	15,568	37.6	179	23.6	15,389	37.8	
25-29	9,885	23.9	191	25.1	9,694	23.8	
30-34	6,904	16.7	132	17.4	6,772	16.7	
35-39	4,655	11.2	117	15.4	4,538	11.2	
40+	4,430	10.7	141	18.6	4,289	10.5	
Sex							<0.001
Male	37,393	90.2	647	85.1	36,746	90.3	
Female	4,049	9.8	113	14.9	3,936	9.7	
Service branch							<0.001
Navy	17,060	41.2	374	49.2	16,686	41	
Marine Corps	24,382	58.8	386	50.8	23,996	59	
Rank							0.950
Enlisted	34,482	83.2	633	83.3	33,849	83.2	
Officer	6,960	16.8	127	16.7	6,833	16.8	
Component							<0.001
Active duty	33,017	79.7	530	69.7	32,487	79.9	
Reserve, National Guard	8,425	20.3	230	30.3	8,195	20.1	
PTSD positive screen							<0.001
No	39,548	95.4	550	72.4	38,998	95.9	
Yes	1,894	4.6	210	27.6	1,684	4.1	
NMR							<0.001
No	39,895	96.3	701	92.2	39,194	96.3	
Yes	1,547	3.7	59	7.8	1,488	3.7	
Time between PDHA and PHA, d		mean (SD)		mean (SD)		mean (SD)	
	239	150	231	154	240	150	0.119

Abbreviations: TBI, traumatic brain injury; n , No., number; y, years; PTSD, post-traumatic stress disorder; NMR, not medically ready; PDHA, Post-Deployment Health Assessment; PHA, Periodic Health Assessment; SD, standard deviation; d, days.

**Table 2:** Multivariable Logistic Regression Examining Not Medically Ready Disposition with Traumatic Brain Injury and Other Covariates

Characteristic	OR	95% CI	*P*-value
TBI positive screen			0.003
No	Reference	-	-
Yes	1.5	1.2-2.0	
Age, y			0.002
18-24	Reference	-	-
25-29	0.9	0.8-1.0	
30-34	0.9	0.8-1.1	
35-39	0.8	0.6-0.9	
40+	1.1	0.9-1.4	
Sex			<0.001
Male	Reference	-	-
Female	1.9	1.6-2.2	
Service			0.268
Navy	Reference	-	-
Marine Corps	0.9	0.8-1.1	
Rank			<0.001
Enlisted	Reference	-	-
Officer	0.7	0.6-0.8	
Component			<0.001
Active duty	Reference	-	-
Reserve, National Guard	2.0	1.8-2.2	
PTSD positive screen			<0.001
No	Reference	-	-
Yes	2.5	2.1-2.9	
Time between PDHA and PHA	1.0	1.0-1.0	<0.001

Abbreviations: OR, odds ratio; CI, confidence interval; TBI, traumatic brain injury; y, years; PTSD, post-traumatic brain injury; PDHA, Post-Deployment Health Assessment; PHA, Periodic Health Assessment.

## AUTHOR AFFILIATIONS

Naval Health Research Center, San Diego: Dr. MacGregor, Ms. Dougherty, Mr. Zouris, Dr. Jurick; Leidos, Inc., San Diego: Ms. Dougherty, Dr. Jurick

## DISCLAIMER

This work was prepared by employees of the U.S. Government as part of official duties. Title 17, U.S.C. §105 provides that copyright protection under this title is not available for any work of the U.S. Government. Title 17, U.S.C. §101 defines a U.S. Government work as work prepared by a military service member or employee of the U.S. Government as part of that individual’s official duties. This report was supported by the U.S. Navy Bureau of Medicine and Surgery under work unit 60808. The views expressed in this article are those of the authors and do not necessarily reflect the official policy nor position of the U.S. Department of the Navy, Department of Defense, or the U.S. Government. The study protocol was approved by the Naval Health Research Center Institutional Review Board in compliance with all applicable Federal regulations governing the protection of human subjects. Research data were derived from an approved Naval Health Research Center Institutional Review Board protocol NHRC.2003.0025.
